# Antifungal Combination of Ethyl Acetate Extract of *Poincianella pluviosa* (DC.) L. P. Queiros Stem Bark With Amphotericin B in *Cryptococcus neoformans*

**DOI:** 10.3389/fmicb.2021.660645

**Published:** 2021-06-10

**Authors:** Gabriella Maria Andriani, Ana Elisa Belotto Morguette, Laís Fernanda Almeida Spoladori, Patrícia Morais Lopes Pereira, Weslei Roberto Correia Cabral, Bruna Terci Fernandes, Eliandro Reis Tavares, Ricardo Sérgio Almeida, Cesar Armando Contreras Lancheros, Celso Vataru Nakamura, João Carlos Palazzo Mello, Lucy Megumi Yamauchi, Sueli Fumie Yamada-Ogatta

**Affiliations:** ^1^Programa de Pós-graduação em Microbiologia, Departamento de Microbiologia, Centro de Ciências Biológicas, Universidade Estadual de Londrina, Londrina, Brazil; ^2^Laboratório de Biologia Molecular de Microrganismos, Departamento de Microbiologia, Centro de Ciências Biológicas, Universidade Estadual de Londrina, Londrina, Brazil; ^3^Programa Nacional de Pós-Doutorado, CAPES, Londrina, Brazil; ^4^Laboratório de Inovação Tecnológica no Desenvolvimento de Fármacos e Cosméticos, Departamento de Ciências Básicas da Saúde, Centro de Ciências da Saúde, Universidade Estadual de Maringá, Maringá, Brazil; ^5^Laboratório de Biologia Farmacêutica, Departamento de Farmácia, Universidade Estadual de Maringá, Maringá, Brazil

**Keywords:** antibiofilm, antivirulence, *Caesalpinia pluviosa*, cryptococcosis, *Galleria mellonella*, synergism

## Abstract

*Cryptococcus neoformans* is the leading cause of cryptococcosis, an invasive and potentially fatal infectious disease. Therapeutic failures are due to the increase in antifungal resistance, the adverse effects of drugs, and the unavailability of therapeutic regimens in low-income countries, which limit the treatment of cryptococcosis, increasing the morbidity and mortality associated with these infections. Thus, new antifungal drugs and innovative strategies for the cryptococcosis treatment are urgently needed. The aim of the present study was to evaluate the effect of ethyl acetate fraction (EAF) of *Poincianella pluviosa* stem bark on planktonic and biofilm mode of growth of *C. neoformans*. Furthermore, the interaction between the EAF and amphotericin B (AmB) was evaluated *in vitro* and in *Galleria mellonella* infection model. Minimal inhibitory concentrations (MICs) of EAF ranged from 125.0 to >1,000.0 μg/ml and >1,000.0 μg/ml for planktonic and sessile cells, respectively. The combination between EAF and AmB exhibited a synergistic fungicidal activity toward *C. neoformans*, with a fractional inhibitory concentration index (FICI) ranging from 0.03 to 0.06 and 0.08 to 0.28 for planktonic and sessile cells, respectively. Microscopy analyses of planktonic *C. neoformans* cells treated with EAF, alone or combined with AmB, revealed morphological and ultrastructural alterations, including loss of integrity of the cell wall and cell membrane detachment, suggesting leakage of intracellular content, reduction of capsule size, and presence of vacuoles. Moreover, EAF alone or combined with AmB prolonged the survival rate of *C. neoformans*-infected *G. mellonella* larvae. These findings indicate that *P. pluviosa* may be an important source of new compounds that can be used as a fungus-specific adjuvant for the treatment of cryptococcosis.

## Introduction

Cryptococcosis is a potentially fatal fungal infection caused mainly by species of the *Cryptococcus gattii* and *Cryptococcus neoformans* complexes (Maziarz and Perfect, 2016). Currently, cryptococcosis ranks as the second most prevalent disease in human immunodeficiency virus (HIV)-infected individuals, with approximately 223,100 new cases and 181,000 deaths per year, particularly in low- and middle-income countries (WHO, 2018). The main contributors to the high mortality associated with cryptococcosis in these countries include delayed diagnosis, limited access and high cost of the drugs used in the induction phase and difficulty in monitoring drug toxicity (WHO, 2018). In fact, the etiological treatment of cryptococcosis is of paramount importance to reduce the mortality rate of this disease, and the use of a potent fungicidal agent during the induction (initial) phase is highly recommended (Maziarz and Perfect, 2016; Bermas and Geddes-McAlister, 2020). However, antifungals commonly used for induction and maintenance therapy schemes exhibit toxicity due to prolonged use, which is generally required (Bermas and Geddes-McAlister, 2020).

Clinically, cryptococcal meningitis is the most common presentation of cryptococcosis, followed by pulmonary cryptococcosis. Moreover, skin, lymph node, and bone involvement can also occur (Maziarz and Perfect, 2016). The etiological treatment of cryptococcosis is based on and limited to the use (alone or combined) of the polyene amphotericin B (AmB), azoles derivatives (mainly fluconazole), and the pyrimidine analog flucytosine, whose treatment regimens depend on the clinical presentation and the immune status of the patient (Perfect et al., 2010; Bermas and Geddes-McAlister, 2020).

Amphotericin B, a fungicidal agent, is considered the gold standard for the treatment of disseminated fungal infections (WHO, 2018). Although antifungal resistance to this drug is rare among cryptococcal isolates, cases of therapeutic failure have been documented (Singhal et al., 2016; Bandaranayake et al., 2018). Furthermore, prolonged treatment with AmB can lead to renal failure, hypokalemia, hypomagnesemia, and anemia (Bermas and Geddes-McAlister, 2020). Fluconazole, a fungistatic agent, is the second line for cryptococcosis treatment. Although it is a well-tolerated drug, gastrointestinal symptoms are frequently reported as adverse effects (Govindarajan et al., 2020). Unlike AmB, prophylactic or subinhibitory doses of fluconazole have led to the selection of resistant cryptococcal isolates, as well as being associated with the recurrence of infections (Perfect et al., 2010; Cheong and McCormack, 2013). Regarding flucytosine, the major drawback is related to the frequent development of resistance, so it is always used combined with another antifungal agent. Moreover, hematological and hepatic toxicities are adverse effects related to this drug (Padda and Parmar, 2020).

Due to this critical scenario and the fact that new classes of antifungals have not been made available by the pharmaceutical industry over the past two decades, there is an urgent need in researching and developing new drugs or strategies that are effective, safe, and affordable for the treatment of cryptococcosis. Thus, in recent decades, we have seen renewed interest in active compounds isolated from natural products, especially plants. In fact, plants are a source of different chemical classes of biologically active molecules, many of which have been proven to present antimicrobial activities against different microorganisms (Biasi-Garbin et al., 2016; Kokoska et al., 2018; Morguette et al., 2019).

Brazilian biomes exhibit a wide biodiversity of native or exotic flora and a high capacity for their sustainable exploitation, and many plants are used in folk medicine to treat different diseases (Savi et al., 2019; Ribeiro Neto et al., 2020). The *Caesalpinia* genus (Fabaceae family) consists of more than 500 species, of which only about 30 species have been studied. Various biological activities have been attributed to extracts or phytochemicals obtained from different species of *Caesalpinia*, such as antimicrobial, anti-inflammatory, antioxidant, anticancer, antidiabetic, antirheumatic, and analgesic activities (Zanin et al., 2012). *Poincianella pluviosa* (DC.) L. P. Queiros [also named *Caesalpinia pluviosa* DC. var. *peltophoroides* (Benth.) G. P. Lewis], popularly known as “sibipiruna” or “falso pau brasil,” is a domesticated plant in Brazil found mainly in the Atlantic forest and Pantanal biomes; it is widely used in ornamentation and is known for its high wood potential (Carvalho, 2014). Few studies on the pharmacological activities of *P. pluviosa* are described in the literature, and the following activities have been specifically attributed to stem bark extract of *P. pluviosa*, including antimalarial (Deharo et al., 2001; Kayano et al., 2011), anti-staphylococcal (Guidi et al., 2020), wound healing *in vitro* and *in vivo* (Bueno et al., 2016; Guidi et al., 2020), and anti-inflammatory (Domingos et al., 2019).

The combination of two or more drugs that generate synergistic effects is another strategy that has been explored for the treatment of fungal infections (Longhi et al., 2016). This strategy may result in lower drug concentrations to produce an effect; reduction of the selection of resistant strains, thus increasing the efficacy of the treatment; and reduction of its toxicity by neutralizing or eliminating adverse effects (Mukherjee et al., 2005; Chen et al., 2014). Currently, studies on synergistic interactions of natural products with clinically important antimicrobial agents have been carried out and are increasingly promising in the formulation of new therapeutic strategies (Chen et al., 2014; Longhi et al., 2016).

Therefore, the aim of the present study was to evaluate the antifungal potential of *P. pluviosa* stem bark extract and its combination with AmB on *C. neoformans*.

## Materials and Methods

### Microorganisms and Culture Conditions

*Cryptococcus neoformans* serotype A ATCC 34872, *C. neoformans* serotype D ATCC 66031, and four isolates of *C. neoformans* (Table 1) recovered from human infections and belonging to the microbial collection of the Laboratory of Molecular Biology of the Microorganisms, Universidade Estadual de Londrina, Londrina, Brazil, were included in the present study. Yeasts were cultured in Sabouraud dextrose (SD) agar at 37°C and kept at 4°C. The clinical isolates were identified by PCR using specific primers complementary to intergenic spacer 1 (IGS1) of ribosomal DNA (Tavares et al., 2016). All fungal strains were also stored in SD broth containing 20% glycerol at −80°C. For the experiments, three to five colonies were transferred to SD broth and incubated at 37°C for 48 h. Cells were then centrifuged (10,000 × *g*, for 3 min) and resuspended in 0.85% NaCl solution (saline) to achieve a turbidity equivalent to 0.5 McFarland standard using the DensiCHEK^TM^ PLUS colorimeter (bioMérieux), which corresponded to approximately 1.0–2.0 × 10^6^ colony-forming unit (CFU)/ml (standard fungal suspension). Each standard fungal suspension was then diluted in culture medium to achieve the cell density (inoculum) used in each assay, unless specified.

**TABLE 1:** Minimal inhibitory concentration (MIC*) of *Poincianella pluviosa* stem bark extracts and amphotericin B against *Cryptococcus neoformans.*

**Microorganism**	**Crude extract**	**Ethyl acetate fraction**	**Amphotericin B**
*C. neoformans* ATCC 34872	>1,000.0	1,000.0	0.125
*C. neoformans* ATCC 66031	>1,000.0	1,000.0	0.125
*C. neoformans* 1172	>1,000.0	>1,000.0	0.250
*C. neoformans* 90889	>1,000.0	>1,000.0	0.250
*C. neoformans* CN01	>1,000.0	>1,000.0	0.125
*C. neoformans* CN12	1,000.0	125.0	0.125

*MIC was determined at the lowest concentration capable to inhibit the visual growth of fungal cells. The results were determined after 72 h of incubation and were expressed in *μ*g/ml.

### *Poincianella pluviosa* Extracts and Antifungals

Bark from *P. pluviosa* (DC.) L. P. Queiros was collected at the Universidade Estadual de Maringá (UEM), and a voucher species was deposited in the herbarium of UEM under the number 12492 HUEM. Access to the botanical material was registered in *Sistema Nacional de Gestão do Patrimônio Genético e do Conhecimento Tradicional Associado* (SISGEN, Brazil) under the number A6DD2D2. Moreover, field studies did not involve endangered or protected plant species. The extracts from stem bark of *P. pluviosa* were prepared according to Bueno et al. (2014). For all antifungal susceptibility assays, crude hydroalcoholic extract (CE) and ethyl acetate (EAF) fraction were dissolved in Roswell Park Memorial Institute 1640 (RPMI, Sigma-Aldrich, Brazil) buffered with 0.164 M 3-(*N*-morpholino) propanesulfonic acid, pH 7.2 (RPMI-MOPS) medium containing 10% dimethyl sulfoxide (DMSO) to obtain a stock solution of 4.0 mg/ml. Stock solution of AmB (1.6 mg/ml; Sigma, Brazil) was diluted in 10% DMSO solution in ultrapure water and maintained at −20°C. DMSO did not exceed 1% in all assays.

### Antifungal Susceptibility Testing on Planktonic Cells

Minimal inhibitory concentrations (MICs) of *P. pluviosa* extracts and AmB were determined by the broth microdilution method according to the Clinical and Laboratory Standards Institute [M27 document A3 (CLSI, 2008)] recommendations. An aliquot (100 μl) of fungal cells (0.5–2.5 × 10^3^ CFU/ml) was added to the wells of 96-well U-bottom microtiter plates (Techno Plastic Products, Switzerland) containing two-fold serial dilutions of *P. pluviosa* extracts (1.95–1,000.0 μg/ml) and AmB (0.031–16.0 μg/ml) in RPMI-MOPS. Wells containing medium or medium plus DMSO 1% and wells without fungal cells were used as growth and sterility control, respectively. *Candida parapsilosis* ATCC 22019 was used as quality control. MIC was defined as the lowest concentration capable of inhibiting visual growth after 72 h of incubation at 37°C in comparison to untreated planktonic cells. Compounds with MIC values >1,000.0 μg/ml were considered inactive (Holetz et al., 2002). Here, 10-μl aliquots from the wells without visible growth were transferred onto SD agar to determine the minimal fungicidal concentration (MFC) (Miles et al., 1938). The plates were incubated at 37°C for 72 h, and MFC was determined as the concentration capable of reducing the CFU counts to zero.

### Checkerboard Microdilution Assay

The antifungal effect of EAF combined with AmB was evaluated using the checkerboard broth microdilution assay in 96-well microtiter plates according to Scott et al. (1995). Two-fold serial dilutions of EAF (0.03–1,000.0 μg/ml) and AmB (0.000001–16.0 μg/ml) were, respectively, added across the plate rows and columns. Subsequently, the fungal inoculum (0.5–2.5 × 10^3^ CFU/ml) was added, and the plates were incubated at 37°C for 72 h. The Fractional Inhibitory Concentration Index (FICI) was determined from the sum of FIC_*EAF*_ and FIC_*AmB*_. The FIC of each compound is the concentration that presents the inhibitory effect when used combined with another compound divided by the concentration that has the same effect when used individually. The FICI values were interpreted as follows: synergism, FICI ≤ 0.5; no interaction, 0.5 < FICI < 4.0; antagonism, FICI > 4.0 (Odds, 2003).

### Characterization of the Synergistic Antifungal Interaction Between Ethyl Acetate Fraction and Amphotericin B

#### Time-Kill Kinetics

The rate of fungal killing in presence of EAF and AmB alone or combined at the MIC values was analyzed by the time-kill assay (CLSI, 2008). Planktonic cells (1.0 × 10^3^ CFU/ml) were added in RPMI-MOPS containing the plant extract and/or the AmB and were incubated statically at 37°C. At specific time points (0, 24, and 48 h), 10 μl were removed from each well and serially diluted (1:10) in 0.15 M phosphate-buffered saline (PBS) pH 7.2. An aliquot of 10 μl of each dilution was inoculated onto SD agar, and the CFU counts were determined after incubation at 37°C for 48 h. Fungal cells incubated in the absence of the EAF and AmB were used as growth control. Data were averaged and plotted as log_10_ CFU/ml vs. time (h).

#### Fungal Cell Viability

Yeast viability was evaluated using the LIVE/DEAD^®^ Yeast Viability Kit (Molecular Probes, Invitrogen) according to the manufacturer’s recommendations. Fungal suspensions (1.0 × 10^7^ CFU/ml) of *C. neoformans* ATCC 66031 and *C. neoformans* CN12 were treated with EAF (1,000.0 and 125.0 μg/ml, respectively), AmB (0.125 μg/ml for both strains), and the combination of EAF and AmB (3.9/0.003 μg/ml for both strains) for 12 h. Afterward, untreated and treated cells were incubated with FUN1^®^ and Calcofluor White^TM^ dyes and analyzed by fluorescence microscopy (LEICA DM2000) using fluorescein filters with excitation/emission of 480/530 nm.

#### Transmission Electron Microscopy (TEM) Analysis of Planktonic Cells

Morphological and ultrastructural changes induced by EAF (1,000.0 μg/ml), AmB (0.125 μg/ml), and the combination of EAF and AmB (3.9 μg/ml and 0.003 μg/ml, respectively) on planktonic cells after 48 h of treatment were analyzed by TEM. Yeast cells were fixed for 2 h at room temperature with 2.5% glutaraldehyde and 4% paraformaldehyde in 0.1 M sodium cacodylate buffer, pH 7.4. Postfixation in 1% OsO_4_ in cacodylate buffer containing 0.8% potassium ferrocyanide and 5 mM CaCl_2_ for 2 h. The cells were then dehydrated in a graded series of acetone and embedded in Epon resin for 72 h at 60°C. Ultrathin sections were obtained with a Leica ultramicrotome, and the sections were contrasted with 5% uranyl acetate and lead citrate for observation in a JEOL JEM-1400 Electron Microscope at 80 kV.

#### Effect of Ethyl Acetate Fraction and Amphotericin B on Capsule and Cell Size

*Cryptococcus neoformans* ATCC 66031 and *C. neoformans* CN12 were cultivated in minimal capsular induction medium (15 mM glucose, 10 mM MgSO_4_, 29.4 mM KH_2_PO_4_, 13 mM glycine, and 3 μM thiamine-HCl, pH 5.5) for 7 days at 30°C (Frases et al., 2009). After incubation, a standard fungal suspension of each strain was prepared as described in section “Microorganisms and Culture Conditions,” and yeast cells (1.0 × 10^7^ CFU/ml) were treated with MIC values of EAF and AmB, alone or in combination at the MIC synergistic concentrations, in RPMI-MOPS for 48 h at 37°C. Untreated cells were used as control. Cells were centrifuged, suspended in Chinese ink, and observed in a Zeiss Axio Imager 2 microscope. Capsule size was measured in 100 cells using the ImageJ 1.49v software^1^. The capsule thickness was determined by the difference between the diameter of the cell, including the capsule, and the diameter of the body cell within the cell wall (Spadari et al., 2018).

#### Effect of Ethyl Acetate Fraction and Amphotericin B on Biofilms

The biofilms were formed on flat-bottomed 96-well polystyrene plates in SD broth according to Martinez and Casadevall (2006) at 37°C for 48 h statically, with an initial inoculum of 1.0 × 10^7^ CFU/ml. After the incubation, non-adherent cells were removed by washing with sterile saline, and 200-μl aliquots of RPMI-MOPS containing different concentrations of EAF (31.25–1,000.0 μg/ml) or AmB (0.007–16.0 μg/ml) were added to the wells for determining the sessile minimal inhibitory concentration (SMIC). The checkerboard assays were used to evaluate the effect of EAF combined with AmB on 48-h biofilm, as described above. Untreated and treated biofilms were incubated at 37°C for 48 h and then washed with sterile saline. The viability of sessile cells was determined by using the dimethylthiazol diphenyltetrazolium bromide (MTT, Sigma) reduction assay according to the manufacturer’s recommendations. The SMICs of EAF and AmB alone and combined were determined by the lowest concentration of the extract/antifungal capable of inhibiting 80% (SCIM_80_) of the sessile cells when compared to the untreated control. The results of the combination were interpreted using the FICI as described above.

#### Scanning Electron Microscopy (SEM) Analysis of Biofilms

Morphological alterations induced by EAF alone and combined with AmB on *C. neoformans* ATCC 66031 biofilm were analyzed by SEM. The polystyrene strips (0.5 cm^2^) and glass (round coverslip) were placed in wells of 24-well tissue culture plates containing 1.0 ml of SD broth, then the biofilm was formed as described above. The biofilms were fixed with 2.5% (v/v) glutaraldehyde in 0.1 M sodium cacodylate buffer pH 7.2 at room temperature and postfixed in 1% OsO_4_, dehydrated with a series of ethanol washes (30, 50, 70, 90, and 100%), critical point dried in CO_2_, coated with gold, and observed in a Shimadzu SS-550 scanning electron microscope.

### Effect of Ethyl Acetate Fraction and Amphotericin B on Mammalian Cells

The cytotoxicity of EAF alone and combined with AmB was evaluated on human erythrocytes. Blood was collected from a healthy donor according to the Declaration of Helsinki principles, and 4% defibrinated blood was prepared in glycosylated saline (0.85% NaCl plus 5% glucose). Erythrocytes (100 μl) were inoculated in each well of 96-well microtiter plates containing different concentrations of EAF (1.95–1,000.0 μg/ml), AmB (0.015–16 μg/ml) alone or in combination. Wells without EAF and AmB and with 1% Triton X-100 were used as negative and positive hemolysis controls, respectively. After incubation for 3 h at 37°C, the optical density (OD) of the supernatant was determined at 550 nm with a microtiter plate reader (Synergy^TM^ HT, BioTek). Thus, the plates were centrifuged at 1,000 × *g* for 10 min, and the supernatants were transferred to new microplates before spectrophotometric reading. The percentage of hemolysis was compared with the positive control wells using the equation: (OD_550_ of the treated supernatant – OD_550_ of the untreated control)/OD_550_ of the positive control – OD_550_ of the untreated control) × 100% (Izumi et al., 2012). The concentration of EAF capable of causing 90% hemolysis was used to calculate the selectivity index (IS) using the following equation: IS = CC_90_/MIC.

### *Galleria mellonella* Infection and Antifungal Treatment

The wax moth larvae killing assays were carried out as described previously with minor modifications (Fuchs et al., 2010). Groups of 10 larvae were used in all assays, and a volume of 5 μl containing the yeast inoculum or antifungals or PBS was inoculated with a Hamilton syringe (Hamilton, United States) into the hemocele. The larva abdomen was cleaned with 70% ethanol before the inoculation. First, the toxicity of EAF and AmB was evaluated by inoculating different concentrations of the compounds per kilogram of larvae as follows: a) 0.25 × MIC, 0.5 × MIC, and MIC values of EAF (*C. neoformans* ATCC 66031 250.0, 500.0, and 1,000.0 μg/ml, respectively; *C. neoformans* CN12 31.2, 62.5, and 125.0 μg/ml, respectively); b) MIC and 2 × MIC of AmB (0.125 and 0.25 μg/ml, respectively, for both strains); c) MIC values of EAF and AmB in the synergistic combination, 2 × MIC and 4 × MIC (3.9/0.003, 7.8/0.006, and 15.6/0.015 μg/ml, respectively, for both strains). All doses were inoculated in the last left proleg of larvae. A group of larvae inoculated with PBS was used as control. For larva infection and treatment, *C. neoformans* ATCC 66031 and *C. neoformans* CN12 were cultivated in minimal capsular induction medium as described in section “Effect of Ethyl Acetate Fraction and Amphotericin B on Capsule and Cell Size.” Fungal cell suspension of each strain was prepared in PBS, and 5 × 10^8^ cells were inoculated into the hemocele in the last left proleg. The treatment with EAF and AmB, alone and combined (as above), was carried out immediately post-infection by inoculating the compounds in the last right proleg. The larvae were incubated at 37°C, and survival was monitored every day up to 10 days. The larvae were considered dead when they did not respond to physical stimulation (slight pressure with forceps). A group of non-infected larvae and a group of infected larvae and treated with PBS were used as controls. Each experiment was carried out in triplicate, and the results presented are from a representative experiment.

### Statistical Analysis

GraphPad Prism version 6.0 software (GraphPad Software, San Diego, CA, United States) was used for statistical analysis. Data of antifungal effect of EAF alone and combined with AmB on capsule and cell size and biofilm were analyzed by one-way ANOVA. The analysis of *G. mellonella* survival data was performed using the log-rank (Mantel–Cox). For all assays, a *p* < 0.05 was considered significant.

## Results

### Crude Extract and Ethyl Acetate Fractions of *Poincianella pluviosa* Do Not Exhibit Antifungal Activity on Planktonic Cells of Most *Cryptococcus neoformans* Strains

To validate the MIC values obtained for EAF against *C. neoformans* strains, MIC values of fluconazole and AmB for the quality control *C. parapsilosis* ATCC 22019 were also determined. The MIC values of fluconazole (1.0 μg/ml) and AmB (0.25 μg/ml) identified for this fungal species were in accordance with CLSI guidelines (CLSI, 2008). The antimicrobial activity of CE and EAF of *P. pluviosa* stem bark was initially evaluated on planktonic cells of *C. neoformans*, and the MIC values are presented in Table 1. MICs ≥1,000.0 μg/ml for the plant compounds were detected for most cryptococcal strains, indicating that they were inactive against planktonic cells. *C. neoformans* CN12 strain was more sensitive to *P. pluviosa* extracts, judging by the MIC values equal to 1,000.0 and 125.0 μg/ml for CE and EAF, respectively. MFC values of EAF were ≥1,000.0 μg/ml for all strains tested, indicating a fungistatic effect.

### Ethyl Acetate Fraction Combined With Amphotericin B Displays Synergistic Interaction Against Planktonic Cells of *Cryptococcus neoformans* Strains

**FIGURE 1:**
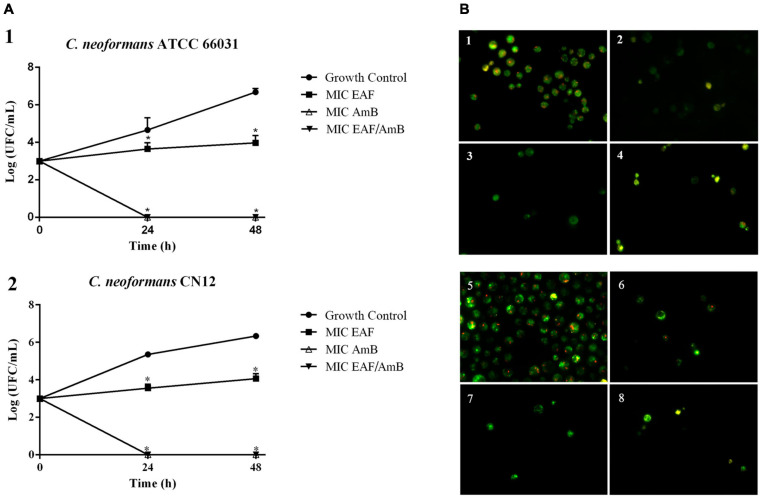
Effect of ethyl acetate fraction (EAF) of *Poincianella pluviosa* bark and amphotericin B (AmB) alone or in combination against *Cryptococcus neoformans*. **(A)** Time-kill kinetics of EAF, AmB, and their combination at 0, 24, 48, and 72 h. **(1)** *C. neoformans* ATCC 66031; **(2)** *C. neoformans* CN12. The values are the mean ± standard deviation of two independent experiments in duplicate. Analysis of *C. neoformans* survival data was performed using two-way ANOVA, *p < 0.05. **(B)** Cell viability analysis of *C. neoformans* ATCC 66031 **(1–4)** and *C. neoformans* CN12 **(5–8)** by fluorescence microscopy using FUN-1^®^ dye. Yeasts were incubated with or without the minimal inhibitory concentrations (MICs) of the two compounds alone or combined for 12 h. Cells with diffuse greenish-yellow fluorescence characterize metabolically inactive cells, and cells containing red fluorescent structures in their vacuoles represent metabolically active yeast. **(1,5)** untreated viable cells; **(2)** 1,000.0 μg/ml EAF; **(3,7)** 0.125 μg/ml AmB; **(4,8)** 3.9/0.003 μg/ml EAF/AmB; **(6)** 125.0 μg/ml EAF.

Based on EAF MIC values, *C. neoformans* ATCC 66031 and *C. neoformans* CN12 were selected for analyzing the effect of simultaneous addition of plant extract and AmB during their planktonic growth by checkerboard assay. Thereafter, both strains were named ATCC 66031 and CN12, respectively. A remarkable reduction in MIC values of EAF and AmB combined was observed; a 32-fold reduction in MIC value of AmB was observed for both strains, whereas a 256-fold and 32-fold reduction in MIC values of EAF were detected for ATCC 66031 and CN12 strains, respectively. The calculated FICI of 0.03 (for ATCC 66031) and 0.06 (for CN12) indicated a synergistic antifungal interaction for the combination of 3.9 μg/ml EAF and 0.003 μg/ml AmB for both strains (Table 2). The results of time-kill studies confirmed this interaction and its fungicidal effect (Figures 1A1,A2). In the presence of MIC values of EAF, an inhibition of planktonic growth of both cryptococcal strains was observed over time compared to untreated control cells. After 48-h incubation, there was a difference of 2 log in CFU counts in EAF-treated cells compared to the untreated ones (*p* < 0.05). At the synergistic combination, the CFU counts of both cryptococcal strains were zero after 24 h, indicating a fungicidal effect. Importantly, this fungicidal effect was similar to that obtained with AmB (MIC) but at lower concentrations of the drug.

**TABLE 2:** Effect of ethyl acetate fraction (EAF) combined with amphotericin B (AmB) against planktonic cells and mature biofilm of *Cryptococcus neoformans.*

**Microorganism**	**EAF (μg/ml)**	**AmB (μg/ml)**	**EAF/AmB (μg/ml)**	**FICI**	**Interaction**
Planktonic cells*					
*C. neoformans* ATCC 66031	1,000.0	0.125	3.9/0.003	0.03	Synergism
*C. neoformans* CN12	125.0	0.125	3.9/0.003	0.05	Synergism
48-h biofilm**					
*C. neoformans* ATCC 66031	>1,000.0	2	31.25/0.5	0.28	Synergism
*C. neoformans* CN12	>1,000.0	4	15.6/0.25	0.07	Synergism

*Minimal inhibitory concentration. **Minimal inhibitory concentration capable to reduce the viability of 80% of sessile cells (SMIC_80_). The results were determined after 72 h of incubation and were expressed in μg/ml. EAF, ethyl acetate fraction; AmB, amphotericin B; EAF/AmB, EAF combined with AmB; FICI, fractional inhibitory concentration index. Reference values: synergism, FICI ≤ 0.5; no interaction, 0.5 < FICI < 4.0; antagonism, FICI ≥ 4.0 (Odds, 2003).

The fungicidal effect of EAF combined with AmB was further supported by the analysis of cell viability of ATCC 66031 and CN12 using fluorescent dyes for differential labeling. The images show that untreated yeasts of both strains exhibited red fluorescent structures in their cytoplasm, indicating metabolically active cells with intact cytoplasmic membranes (Figures 1B1,B5). However, cells treated with EAF MIC exhibited bright, diffuse, green–yellow fluorescence staining, suggesting cells with poor metabolic activity (Figures 1B2,B6). AmB treatment (Figures 1B3,B7) and the combination of both (Figures 1B4,B8) showed diffuse green staining, indicating cell death.

**FIGURE 2:**
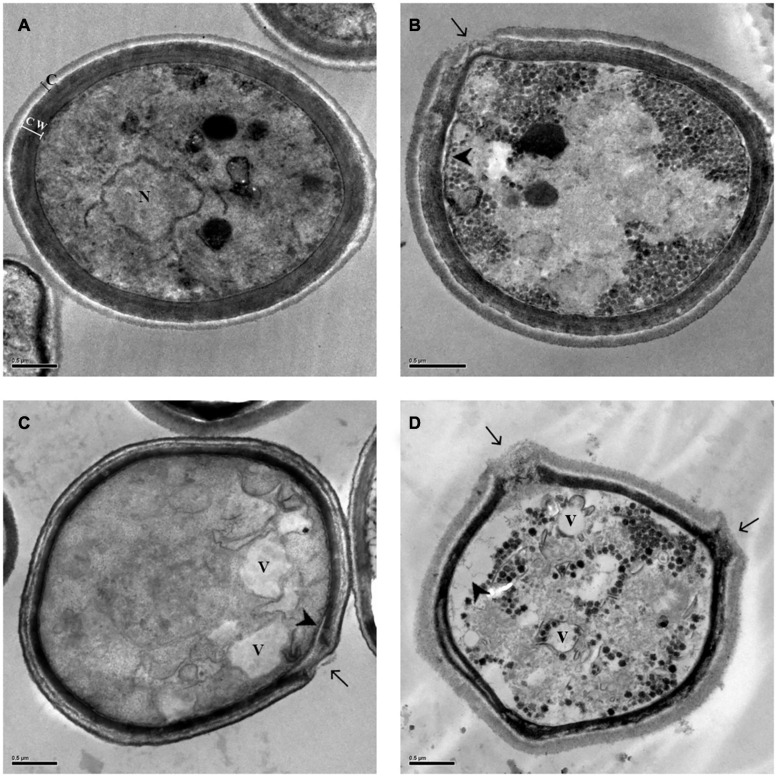
Morphological and ultrastructural changes analyzed by transmission electron microscopy (TEM) in *Cryptococcus neoformans* ATCC 66031 after 48 h of treatment with ethyl acetate fraction (EAF) and amphotericin B (AmB) alone or combined. **(A)** Untreated control cells grown in RPMI-MOPS for 48 h at 37°C; **(B)** 1,000.0 μg/ml EAF; **(C)** 0.125 μg/ml AmB; **(D)** 3.9/0.003 μg/ml EAF/AmB. Capsule (c), cell wall (cw), nucleus (n), vacuole (v), loss of cell wall integrity (arrow), and cell membrane detachment (arrow head).

To corroborate the time-kill and fungal viability analyses, thus, to elucidate the possible mechanism of action of EAF alone and combined with AmB, the ultrastructure of ATCC 66031 was examined by TEM. Untreated yeasts exhibited typical oval morphology with regular and compact cell wall (cw), surrounded by capsule (c), and normal electron density cytoplasm with evident nucleus (n) (Figure 2A). Treated cells showed significant morphological and ultrastructural alterations. The most observed changes in the treatment with EAF alone or combined with AmB were alterations in cryptococcal morphology, including irregularity of the cell wall with loss of integrity, suggesting leakage of intracellular content (arrow, Figures 2B–D) and cell membrane detachment (arrowhead, Figures 2B–D). Decreased electron density and presence of vacuoles were also observed in the treatment with the plant extract alone or combined with AmB (V, in Figures 2C,D).

**FIGURE 3:**
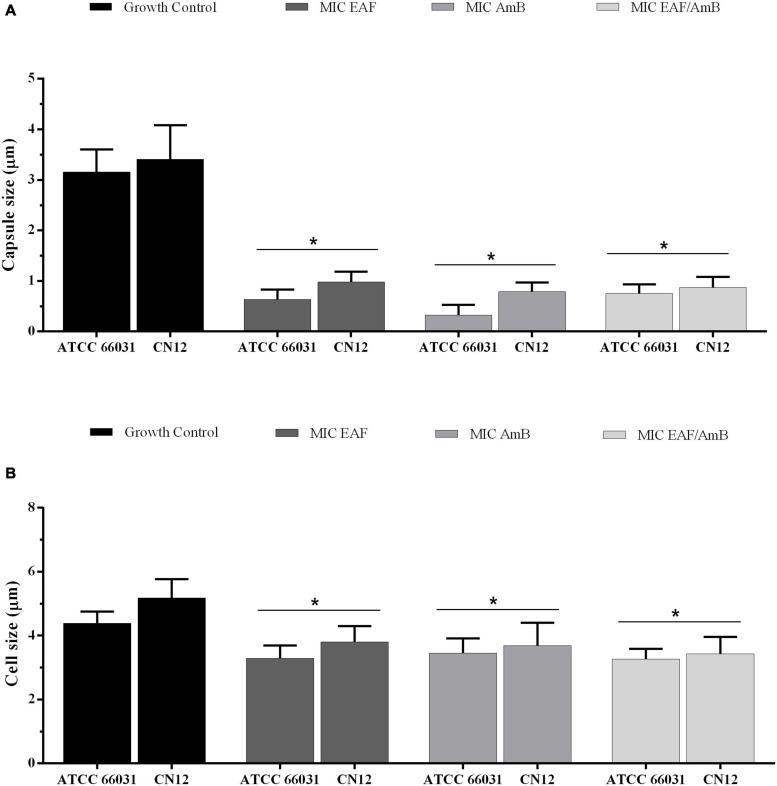
Effect of ethyl acetate fraction (EAF) of *Poincianella pluviosa* bark and amphotericin B (AmB) alone or in combination on capsule **(A)** and cell **(B)** size. *Cryptococcus neoformans* ATCC 66031 and *C. neoformans* CN12 were treated with EAF minimal inhibitory concentration (MIC) (1,000.0 and 125 μg/ml, respectively), AmB MIC (0.125 μg/ml), and EAF combined with AmB (3.9/0.003 μg/ml), and the results were compared to untreated control. A total of 100 cells were measured, and the mean ± standard deviation was calculated and analyzed by one-way ANOVA. Asterisks indicate a significant reduction (*p* < 0.05) in the metabolic activity of treated sessile cells compared to untreated cells.

To observe the effect of the EAF alone or combined with AmB on capsule, the thickness of this structure was measured using light microscopy in yeast cells negatively stained with Chinese ink. Untreated cells were typically round and were surrounded by a clear capsule (Supplementary Figure 1). Treatments with EAF and AmB, alone or in combination, lead to a significant (*p* < 0.05) reduction in capsule (Figure 3A) and cell size (Figure 3B) after 48 h of incubation when compared to the untreated control cells of both cryptococcal strains.

### Ethyl Acetate Fraction Alone or Combined With Amphotericin B Exhibits Antibiofilm Activity in *Cryptococcus neoformans*

**FIGURE 4:**
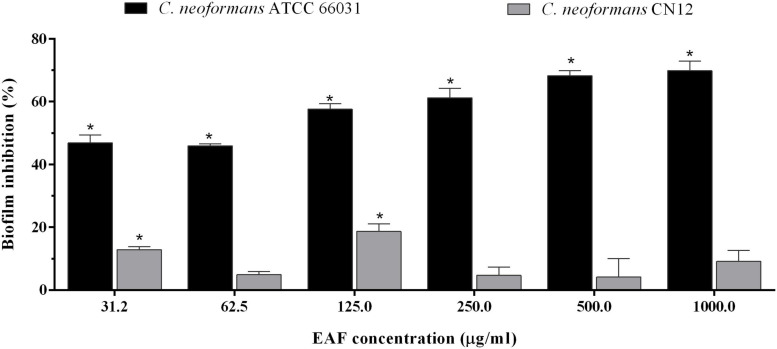
Effect of ethyl acetate fraction (EAF) of *Poincianella pluviosa* on 48-h biofilm of *Cryptococcus neoformans*. Metabolic activity of sessile cells was assessed by the dimethylthiazol diphenyltetrazolium bromide (MTT) reduction method after 48-h incubation at 37°C with different concentrations of EAF. Values are mean ± standard deviation of two experiments in quintuplicate and were analyzed by one-way ANOVA. Asterisks indicate a significant reduction (*p* < 0.05) in the metabolic activity of treated sessile cells compared to untreated cells.

In addition to the antifungal effect on planktonic cells, EAF combined with AmB exhibited an inhibitory activity on 48-h biofilms of *C. neoformans*. The SMIC_80_ of EAF alone was >1,000.0 μg/ml, as at this concentration, a percentage reduction of 69.8 and 9.1% in sessile cell viability was observed for ATCC 66031 and CN12 strains, respectively (Figure 4 and Supplementary Table 1). Regarding AmB, the SMIC_80_ values of 2.0 and 4.0 μg/ml were identified for ATCC 66031 and CN12 strains, respectively (Table 2). The simultaneous addition of EAF and AmB on 48-h biofilm provoked a significant reduction in MIC values of these compounds for both strains. For ATCC 66031, 32-fold and four-fold reductions in MIC values of EAF and AmB were, respectively, observed; for CN12, 64-fold and 16-fold reductions, respectively. Synergistic antifungal effect was observed on 48-h biofilm of *C. neoformans* strains, with calculated FICI values of 0.28 (ATCC 66031) and 0.08 (CN12) (Table 2).

**FIGURE 5:**
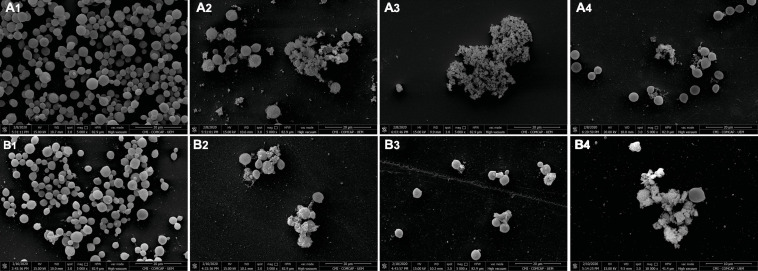
Scanning electron microscopy (SEM) images of *Cryptococcus neoformans* ATCC 66031 biofilms on glass **(A1–A4)** and polystyrene **(B1–B4)** surfaces over 48 h of incubation at 37°C. **(1)** Untreated control; **(2)** 1,000.0 μg/ml ethyl acetate fraction (EAF); **(3)** 2.0 μg/ml amphotericin B (AmB); **(4)** 31.25/0.5 μg/ml EAF/AmB.

Scanning electron microscopy (SEM) images showed the untreated and treated biofilms of ATCC 66031 formed on glass (Figures 5A1–A4) and polystyrene (Figures 5B1–B4) surfaces. On both surfaces, untreated biofilms (Figures 5A1,B1) consisted of cells firmly adhered to the surfaces, exhibiting typical spherical morphology after 48-h incubation. In contrast, a remarkable decrease in the number of cells within the biofilms treated with EAF (Figures 5A2,B2), AmB (Figures 5A3,B3), and their combination (Figures 5A4,B4) was visualized, which was consistent with the reduction of biofilm viability. Moreover, it was possible to observe severe damage with deformed cells and cell debris, indicating cell death (Figures 5A2–A4,B2–B4).

### Ethyl Acetate Fraction Combined With Amphotericin B Does Not Induce Hemolysis on Human Erythrocytes

**FIGURE 6:**
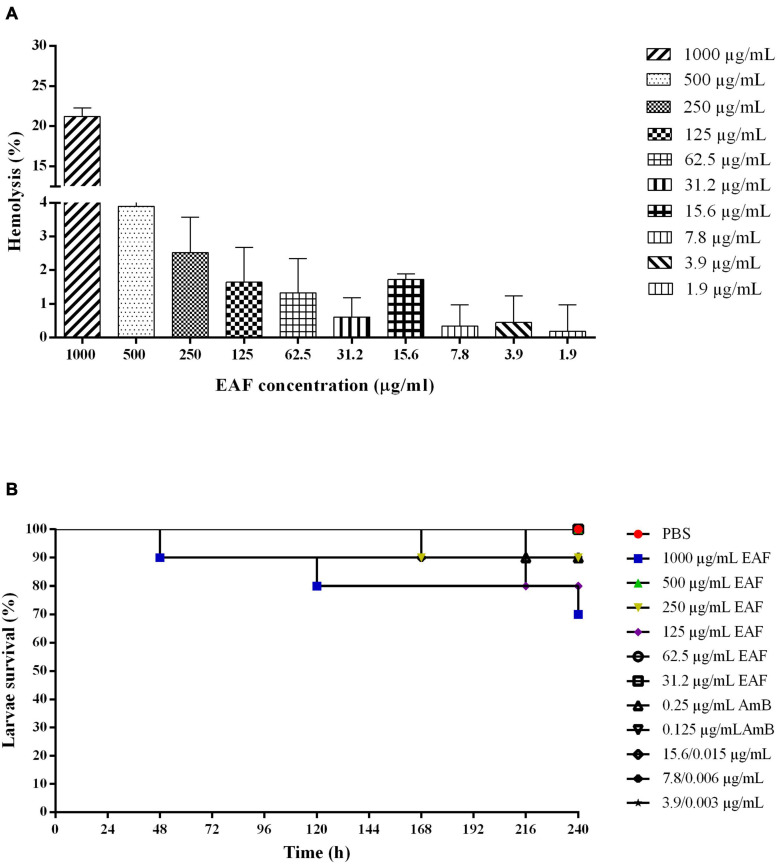
Effect of ethyl acetate fraction (EAF) of *Poincianella pluviosa* bark and amphotericin B (AmB) alone or in combination against human erythrocytes **(A)** and *Galleria mellonella* larvae **(B)**. **(A)** Erythrocytes were treated with different concentrations (1,000.0–1.9 μg/ml) of EAF during 3 h at 37°C. The percentage hemolysis was calculated using untreated cells as control. Values are mean ± standard deviation of two experiments in duplicate. **(B)** Kaplan–Meier plots of survival curves of *G. mellonella* larvae treated with different concentrations of EAF (1,000.0–31.2 μg/kg of larvae), AmB (0.25 or 0.125 μg/kg of larvae), and EAF combined with AmB (15.6/0.015, 7.8/0.006, or 3.9/0.003 μg/kg of larvae) at the synergistic concentrations. Analysis of *G. mellonella* survival data was performed using the log-rank (Mantel–Cox) of representative experiment. PBS, phosphate-buffered saline.

The effect of EAF alone and combined with AmB was evaluated in human erythrocytes. EAF in concentrations ranging from 1.95 to 500.0 μg/ml showed a percentage of hemolysis from 0.1 to 3% and were considered non-hemolytic (Figure 6A). However, at the highest concentration tested (1,000.0 μg/ml), a percentage of hemolysis of 21.2% was detected, indicating that the plant extract may induce erythrocyte lysis in higher concentrations. The combination of different concentrations of EAF and AmB caused around 3.0% hemolysis and were considered non-hemolytic. As it was not possible to calculate the CC_90_ of EAF on human erythrocytes, the highest concentration was used to calculate the IS. Thus, IS values greater than 8 were estimated for CN12 strain, while for ATCC 66031 and the other strains, the value was greater than 1, indicating that EAF may be more toxic toward the fungal species.

### Ethyl Acetate Fraction Alone or Combined With Amphotericin B Does Not Exhibit Toxicity to *Galleria mellonella* Larvae and Reduces the Mortality of the Larvae Infected With *Cryptococcus neoformans*

Based on the MIC values and the results from mammalian cell tests, all treatments of the *G. mellonella* larvae were carried out using different concentrations of the compounds per kilogram of larvae, i.e., 0.25 × MIC, 0.5 × MIC, and MIC values of EAF; MIC and 2 × MIC of AmB; MIC values of EAF and AmB (at the synergistic combination), 2 × MIC and 4 × MIC.

First, the *G. mellonella* larvae were inoculated with EAF and AmB, alone and in combination, to determine the toxicity of the plant extract/antifungal for the larvae. Similar to the control group treated with PBS, a survival rate of 100% was observed with most EAF/AmB treatments after 10 days. Survival rates of 70, 80, and 90% were observed for 1,000.0 μg/ml/kg EAF, 125.0 μg/ml/kg EAF, and 250.0 μg/ml/kg EAF and 0.25 μg/ml/kg AmB, respectively (Figure 6B). Considering these results, the efficacy of these compounds was evaluated in *G. mellonella* infected with ATCC 66031 and CN12 strains.

**FIGURE 7:**
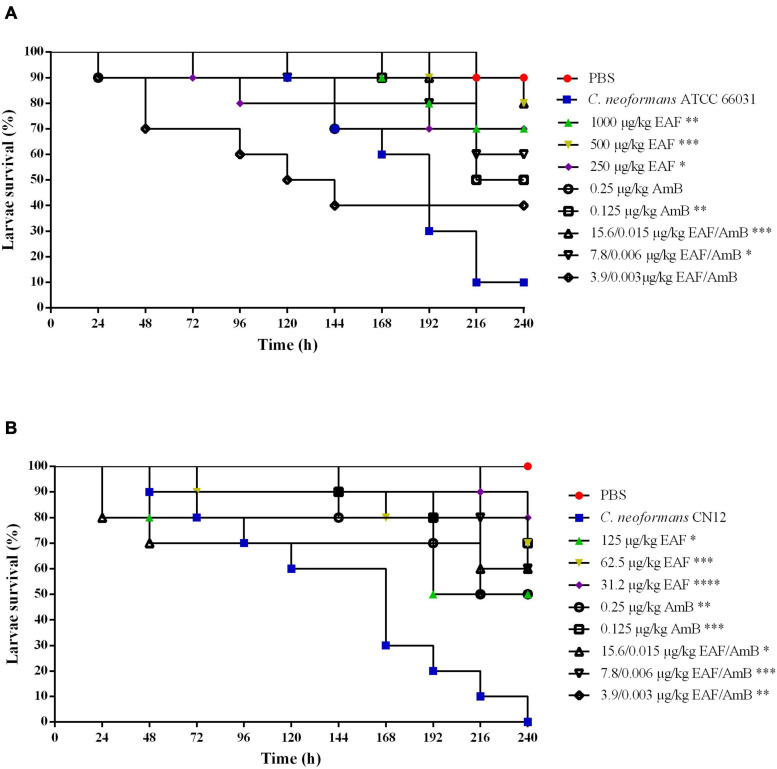
Effect of ethyl acetate fraction (EAF) of *Poincianella pluviosa* bark and amphotericin B (AmB) alone or in combination against *Galleria mellonella* larvae infected with *Cryptococcus neoformans* ATCC 66031 **(A)** and *Cryptococcus neoformans* CN12 **(B)**. All groups were compared with infected and untreated larvae. Analysis of *G. mellonella* survival data was performed using the log-rank (Mantel–Cox) of representative experiment. The asterisks indicate a significant reduction in mortality rate of infected and treated group compared with the infected and untreated group (*p < 0.05; **p < 0.01; ***p < 0.001; ****p < 0.0001). **(A)** Kaplan–Meier plots of survival curves of *G. mellonella* larvae infected with *Cryptococcus neoformans* ATCC 66031. The larvae were infected with fungal cells and concomitantly treated with minimal inhibitory concentration (MIC), 0.5 × MIC, or 0.25 × MIC of EAF (1,000.0, 500.0, or 250.0 μg/ml/kg of larvae, respectively), 2 × MIC and MIC AmB (0.25 or 0.125 μg/ml/kg of larvae, respectively), and EAF combined with AmB (15.6/0.015, 7.8/0.006, or 3.9/0.003 μg/ml/kg of larvae) at the synergistic concentrations. **(B)** Kaplan–Meier plots of survival curves of *G. mellonella* larvae infected with *C. neoformans* CN12. The larvae were infected with fungal cells and concomitantly treated with MIC, 0.5 × MIC, or 0.25 × MIC of EAF (125.0, 62.5, or 31.2 μg/kg of larvae, respectively), AmB (0.25 or 0.125 μg/kg of larvae, respectively), and EAF combined with AmB (15.6/0.015, 7.8/0.006, or 3.9/0.003 μg/kg of larvae) at the synergistic concentrations.

After 168-h post-infection with ATCC 66031, the mortality rate of infected and untreated larvae was 40%, which progressively increased over 240 h, resulting in 90% of mortality (Figure 7A). Treatment with AmB resulted in 50% survival rate of the larvae in both concentrations at the end of the experiment. Interestingly, the treatment of larvae with EAF significantly increased their survival rate in the three concentrations tested compared to the untreated group. After 240 h, 70% (*p* < 0.01), 80% (*p* < 0.001), and 70% (*p* < 0.05) of live larvae were observed for the treatment with MIC, 0.5 × MIC, and 0.25 × MIC of EAF, respectively. The EAF (15.6 μg/ml/kg) combined with AmB (0.015 μg/ml/kg) (4 × MIC) significantly (*p* < 0.001) prolonged larva survival compared to the untreated group (80%, EAF-treated vs. 10%, untreated). Furthermore, this survival rate was greater than those of the AmB (for both doses) and MIC EAF treatments. The combination 7.8 μg/ml/kg EAF with 0.006 μg/ml/kg AmB (2 × MIC) also significantly (*p* < 0.05) prolonged larva survival, with 60% of live larvae, being a better treatment than AmB as well. The other EAF/AmB combination (3.9/0.003 μg/ml) resulted in 40% survival rate (Figure 7A).

All larvae infected with CN12 and untreated died after 240 h of infection (100% mortality rate; Figure 7B). Treatment with AmB resulted in a survival rate of 50% (*p* < 0.01) for 2 × MIC and of 70% (*p* < 0.001) for MIC. EAF treatment was effective in all concentrations tested compared to the untreated group. At the end of the experiment, MIC, 0.5 × MIC, and 0.25 × MIC led to a survival rate of 50% (*p* < 0.05), 70% (*p* < 0.001), and 80% (*p* < 0.0001), respectively. Unlike the infection with ATCC 66031, treatment with all EAF and AmB combinations induced an increase in CN12-infected larvae survival rates compared to untreated larvae. After the treatment with the combination of 4 × MIC (*p* < 0.05) and 2 × MIC (*p* < 0.001), 60% of live larvae were identified, which was a better treatment than 2 × MIC of AmB. At the MIC combination, 50% survival rate (*p* < 0.01) was observed.

## Discussion

The combination therapy has been used to improve the efficacy of drugs and to reduce their adverse effects (Chen et al., 2014), and this strategy has been widely applied for the treatment of different diseases, including those caused by microbial pathogens (Brennan-Krohn and Kirby, 2019). Indeed, the combination of AmB and 5-flucytosine is the recommended antifungal therapy for the treatment of cryptococcal meningitis (Perfect et al., 2010; Bermas and Geddes-McAlister, 2020).

The antifungal potential of *Poincianella* (*Caesalpinia*) species has been previously described. Different parts of *Caesalpinia sappan* (Niranjan Reddy et al., 2003), *Caesalpinia pyramidalis* (Cruz et al., 2007), *Caesalpinia bonducella* (Shukla et al., 2011), *Caesalpinia ferrea* Martius (Macêdo et al., 2020), and *Caesalpinia pulcherrima* (de Melo et al., 2020) showed antifungal effect *in vitro* against planktonic cells of several fungal species, which can cause infections in humans such as *Trichophyton rubrum*, *Candida guilliermondii*, *Candida albicans*, *Candida parapsilosis*, and *Fonsecaea pedrosoi*. Among these plant species, the inhibitory activity of only two species was evaluated on growth of planktonic cells of *C. neoformans*. The aqueous extract (water infusion) of leaves from *C. pyramidalis* inhibited the growth of *C. neoformans* T_1_-444, a clinical isolate, displaying a MIC of 12.5 μg/ml (Cruz et al., 2007). Lignin extracted from the leaves of *C. pulcherrima* inhibited the growth of three *C. neoformans* strains (HC43, HC44, and HC47) with MIC of 15.6 μg/ml (de Melo et al., 2020).

In the present study, the EAF obtained from *P. pluviosa* stem bark exhibited low intrinsic antifungal activity on planktonic cells of *C. neoformans*, presenting a fungistatic effect. However, EAF acted synergistically in combination with AmB, inhibiting the growth of planktonic and viability of sessile (biofilm) cells of this fungal species. Importantly, the fungicidal effect was maintained at lower concentrations of AmB (32-fold and at least four-fold lower for planktonic and sessile cells, respectively) and similar to those death kinetic produced by AmB alone. The combined antifungal effect on 48-h biofilm is an important finding of the present study, since the biofilm formation ability of *Cryptococcus* species is associated with both fungal virulence and reduced susceptibility to antifungals (Martinez and Casadevall, 2015; Benaducci et al., 2016; Tavares et al., 2019), as in other species of several genera of fungi including *Candida*, *Rhodotorula*, *Aspergillus*, *Fusarium*, *Trichosporon*, *Malassezia*, *Histoplasma*, *and Paracocidioides* (Bizerra et al., 2008; Sardi et al., 2014). In fact, the successful eradication of biofilms often requires concentrations of antimicrobial agents that are usually toxic to the host (Sardi et al., 2014; Martinez and Casadevall, 2015). Accordingly, biofilm-related human infections are difficult to treat and have been associated with high mortality rates (Tumbarello et al., 2012). Therefore, the combination of EAF and AmB may be useful for the treatment of biofilm-related cryptococcal infection.

Previous studies reported the antimalarial (Kayano et al., 2011) and anti-staphylococcal (Guidi et al., 2020) activities of the extracts from *P. pluviosa* stem bark. In addition, the combination of *P. pluviosa* extract with artesunate, an artemisinin derivative, reduced the parasitemia of *Plasmodium chabaudi*-infected mice (Kayano et al., 2011); however, there are no reports described for *C. neoformans*.

The mode of action of EAF combined with AmB is unclear. The polyphenol content of EAF from *P. pluviosa* was determined to be 27.98% ± 0.52% (w/w) (Bueno et al., 2012). Flavonoids such as quercetin (Kayano et al., 2011), caesalpinioflavone (Zanin et al., 2015), and hydrolyzable tannins (Bueno et al., 2014) such as pyrogallol, ellagic acid, and gallic acid (Sereia et al., 2019) were identified in the plant extracts, including CE and EAF. Polyphenols are widely distributed in plants, where they participate in various functions related to growth and protection against pathogens, predators, and UV radiation. Several studies indicate that the mechanism by which the plant polyphenols exert their biological activities are due to the ability to bind directly to target proteins, which can affect different processes related to cell function (Quideau et al., 2011). Although data in the literature indicate a possible mechanism of action for EAF, a limitation of our study is that we have not investigated which phytochemical is active against *C. neoformans*. Future studies will be necessary to unveil the active constituent of EAF and its mechanism of action against this fungal species. However, we cannot rule out that the antifungal activity observed in this study can be attributed to the various phytochemicals of *P. pluviosa* EAF acting synergistically with AmB.

Aside from the direct antimicrobial effect, different polyphenolic compounds have been studied for their potential as an adjuvant to clinically used drugs (Zacchino et al., 2017). For instance, epigallocatechin gallate with fluconazole/ketoconazole exhibited synergistic antifungal effect against planktonic and sessile cells of *Candida* spp. *in vitro* (Behbehani et al., 2019). The combination of this polyphenol with AmB significantly decreased the growth of *Candida albicans in vitro* (Hirasawa and Takada, 2004) and increased the survival of mice with disseminated candidiasis caused by this species (Han, 2007). The efficacy of combined therapy *in vivo* was also observed in mice infected with *C. gattii*; although the synergistic effect has not been observed *in vitro*, treatment of infected mice with curcumin and fluconazole reduced and eliminated the fungal burden in lung and brain tissues, respectively, increasing the animals survival (da Silva et al., 2016).

The analysis of the *C. neoformans* ultrastructure by transmission electron microscopy (TEM) revealed that EAF alone or combined with AmB induced important changes in yeast morphology, such as plasma membrane detachment, loss of cell wall integrity causing its rupture, and vacuole formation. Ishida et al. (2009) found similar changes through the exposure of *Cryptococcus* to tannins present in an extract of *Stryphnodendron adstringens*. Treatment with the allylamine terbinafine alone or in combination with fluconazole or AmB also generated similar alterations in the cellular ultrastructure. These changes may be related to altered ergosterols in the plasma membrane, impairing the integrity and function of the cell wall (Guerra et al., 2012).

In the present study, larvae of *G. mellonella* were used to evaluate the efficacy of combined therapy of EAF and AmB *in vivo* through the survival assay. This non-mammalian model can be maintained easily and inexpensively in laboratory conditions, at different temperatures, including the human temperature 37°C. Similarly to mammalian models, this insect develops a specific immune response against different microbial infections (Pereira et al., 2018). Therefore, this insect has been used to study the fungal virulence and pathogenesis (Benaducci et al., 2016; Firacative et al., 2020; Grizante Barião et al., 2020) and to evaluate the efficacy of the antifungal agents against different fungal species (Singulani et al., 2019; de Castro Spadari et al., 2020). The results reported in the present study showed that early treatment with EAF from *P. pluviosa* combined with AmB was effective in controlling *C. neoformans* infection with no toxicity to larvae, supporting the *in vitro* results. The combined therapy (2 × MIC and 4 × MIC) was slightly more effective than monotherapy with both AmB concentrations in larvae infected with ATCC 66031.

Interestingly, EAF monotherapy was the most effective treatment for larvae infected with both strains (survival rates of 80% and 70% with 0.5 × MIC, and 0.25 × MIC and MIC doses for ATCC 66031, respectively; survival rates of 70% and 80% with 0.5 × MIC and 0.25 × MIC doses for CN12, respectively), although a weak inhibitory activity was observed *in vitro*. A significant reduction in capsule size was also observed with the treatment of EAF alone or combined with AmB. Previous studies have shown that AmB treatment reduced the capsule size of *C. neoformans in vitro* (Nosanchuk et al., 1999) and in murine infection (Zaragoza et al., 2005). The polysaccharide capsule is essential for the virulence of *C. neoformans*, contributing to the evasion of immune defenses and survival within the host (Zaragoza et al., 2009). Moreover, it has been shown that the capsule is important for the adhesion of the fungus on abiotic and biotic surfaces, triggering the formation of biofilm (Martinez and Casadevall, 2015; Camacho and Casadevall, 2018). Therefore, the reduction in the size of the *C. neoformans* capsule observed in the present study indicates that the treatment with EAF, alone or combined with AmB, may interfere with the virulence of both planktonic and biofilm cells. Moreover, as described above, hydrolyzable tannins (Bueno et al., 2014) such as pyrogallol, ellagic acid, and gallic acid (Sereia et al., 2019) were identified in the EAF from *P. pluviosa* stem bark. The antifungal activity of all these compounds has already been described for different species of fungi (de Paula E Silva et al., 2014; Yang et al., 2020); besides, the immunomodulatory activity of polyphenols was also well-known (Ding et al., 2018). Particularly, gallic acid inhibits the growth of *C. neoformans* (de Paula E Silva et al., 2014) and can modulate the host innate immune response, increasing defense against microbial infections (Yang et al., 2020). Therefore, the combined antivirulence and immunomodulatory activities of the polyphenols may be responsible for the therapeutic effect of EAF from *P. pluviosa* stem bark observed in *G. mellonella* infection in the present study. Further studies using mammalian models of *C. neoformans* infection should be carried out to support these findings.

In summary, the results of the present study report for the first time the antifungal activity of EAF of *P. pluviosa* stem bark against *C. neoformans*; its combination with AmB exhibited a potent and synergistic antifungal and antivirulence interaction toward *C. neoformans*, reducing significantly the inhibitory concentrations of both compounds for planktonic and sessile cells and preserving the fungicidal activity of AmB. Moreover, EAF alone or combined with AmB prolonged the survival rate of *C. neoformans*-infected *G. mellonella*. Despite the limitations of the present study, the results expand the knowledge about this legume bark’s antimicrobial properties, highlighting the potential of this plant extract for the development of new strategies for the treatment of cryptococcosis.

## Data Availability Statement

The original contributions presented in the study are included in the article/Supplementary Material, further inquiries can be directed to the corresponding author.

## Author Contributions

GA and SY-O performed the conception, experimental design, analysis and interpretation of data, and writing of the manuscript. All authors have read and approved the final manuscript, and have made a substantial methodological and intellectual contribution to the study.

## Conflict of Interest

The authors declare that the research was conducted in the absence of any commercial or financial relationships that could be construed as a potential conflict of interest.

## Footnotes

1 https://imagej.nih.gov/ij/
