# Supplementary material for: Antifungal Combination of Ethyl Acetate Extract of Poincianella pluviosa (DC.) L. P. Queiros Stem Bark With Amphotericin B in Cryptococcus neoformans
Source: Front Microbiol. 2021 Jun 10;12:660645. doi: 10.3389/fmicb.2021.660645 (PMC8222688; doi:10.3389/fmicb.2021.660645)
Supplement: Supplementary file 1 [file Data_Sheet_1.docx]

Supplementary Material

## Supplementary Figures


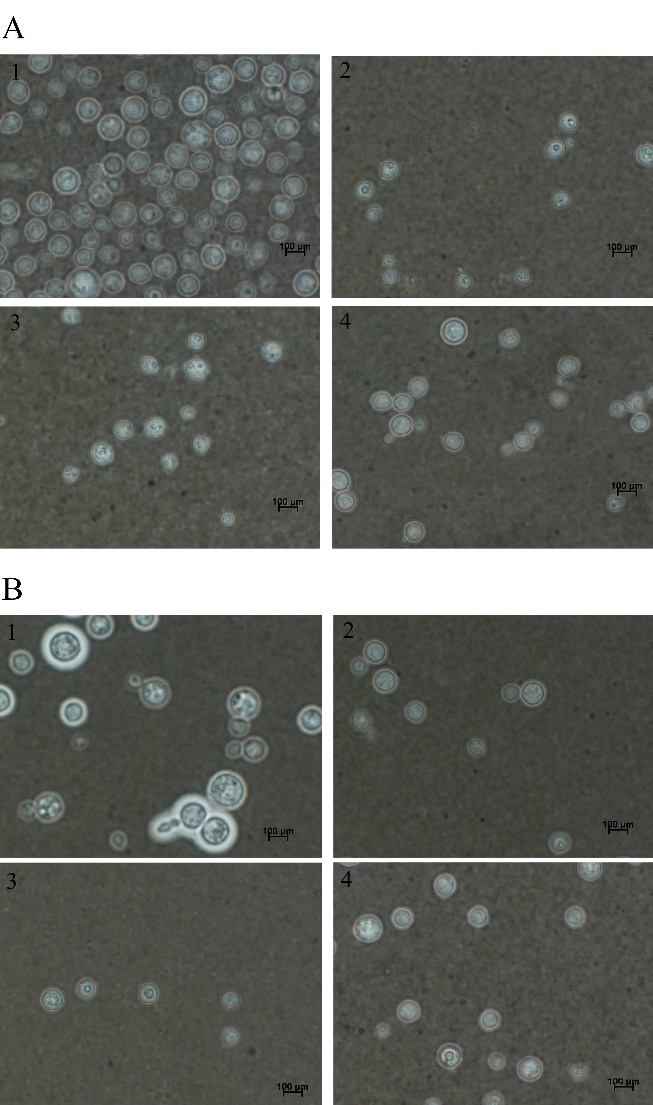


**Supplementary Figure 1.** Effect of ethyl acetate fraction (EAF) of *P. pluviosa* bark and amphotericin B (AmB) alone or combined on capsule and cell size of *C. neoformans* ATCC 66031 (**A**) and *C. neoformans* CN12 (**B**). Yeast cells were negatively stained with Chinese ink and visualized by light microscopy. (**A1** and **B1**) untreated cells; (**A2**) EAF MIC 1000.0 µg/mL; (**B2**) EAF MIC 125 µg/mL; (**A3** and **B3**) AmB MIC (0.125 µg/mL); (**A4** and **B4**) EAF combined with AmB (3.9/0.003 µg/mL).

## Supplementary Tables

**Supplementary Table 1:** Effect of ethyl acetate fraction (EAF) of *P. pluviosa* and amphotericin B (AmB) alone or combined on 48 h-biofilm of *Cryptococcus neoformans*.

| **Treatment (µg/mL)** | **Isolates** | |
| --- | --- | --- |
|  | **ATCC 66031** | **CN12** |
| **Untreated control** | 0.759 ± 0.017 | 0.424 ± 0.094 |
| **EAF** |  |  |
| 1000 | 0.189 ± 0.008 | 0.401 ± 0.101 |
| 500 | 0.218 ± 0.010 | 0.224 ± 0.094 |
| 250 | 0.258 ± 0.026 | 0.355 ± 0.005 |
| 125 | 0.300 ± 0.010 | 0.356 ± 0.089 |
| 62.5 | 0.371 ± 0.012 | 0.345 ± 0.003 |
| 31.2 | 0.495 ± 0.021 | 0.380 ± 0.101 |
| **AmB** |  |  |
| 16 | 0.035 ± 0.003 | 0.022 ± 0.004 |
| 8 | 0.066 ± 0.021 | 0.014 ± 0.002 |
| 4 | 0.109 ± 0.042 | 0.035 ± 0.016 |
| 2 | 0.119 ± 0.049 | 0.053 ± 0.006 |
| 1 | 0.126 ± 0.011 | 0.067 ± 0.024 |
| 0,5 | 0.146 ± 0.024 | 0.164 ± 0.020 |
| **EAF/AmB** |  |  |
| 62.5/1 | 0.096 ± 0.038 | 0.015 ± 0.004 |
| 31.2/0.062 | 0.134 ± 0.062 | 0.030 ± 0.009 |
| 15.6/0.031 | 0.146 ± 0.007 | 0.059 ± 0.022 |
| 7.8/0.015 | 0.135 ± 0.018 | 0.023 ± 0.013 |
| 3.9/0.007 | 0.144 ± 0.013 | 0.037 ± 0.031 |
| 1.9/0.003 | 0.168 ± 0.039 | 0.126 ± 0.095 |

Metabolic activity of sessile cells was assessed by the MTT reduction method after 48 h incubation at 37 °C with different concentrations of EAF and AmB alone or combined. Values were expressed in optical density (O. D.) and are mean ± standard deviation of two experiments in quintuplicate and were analyzed by one way ANOVA. Negative control (without biofilm) was subtracted from all O.D. values to eliminate the background staining interference.
